# Gut Dysbiosis in Irritable Bowel Syndrome: A Narrative Review on Correlation with Disease Subtypes and Novel Therapeutic Implications

**DOI:** 10.3390/microorganisms11102369

**Published:** 2023-09-22

**Authors:** Maria Napolitano, Ernesto Fasulo, Federica Ungaro, Luca Massimino, Emanuele Sinagra, Silvio Danese, Francesco Vito Mandarino

**Affiliations:** 1 Department of Gastroenterology and Gastrointestinal Endoscopy, IRCCS San Raffaele Hospital, 20132 Milan, Italy; fasulo.ernesto@hsr.it (E.F.); ungaro.federica@hsr.it (F.U.); massimino.luca@hsr.it (L.M.); danese.silvio@hsr.it (S.D.); mandarino.francesco@hsr.it (F.V.M.); 2 Division of Immunology, Transplantation and Infectious Disease, IRCCS Ospedale San Raffaele, 20132 Milan, Italy; 3 Gastroenterology & Endoscopy Unit, Fondazione Istituto G. Giglio, Contrada Pietra Pollastra Pisciotto, 90015 Cefalù, Italy; emanuelesinagra83@googlemail.com; 4 Gastroenterology and Endoscopy, Vita-Salute San Raffaele University, 20132 Milan, Italy

**Keywords:** irritable bowel syndrome, IBS, microbiota, microflora, gut, subtype IBS-D, subtype IBS-C

## Abstract

Irritable bowel syndrome (IBS) is a prevalent functional gastrointestinal disorder characterized by chronic abdominal pain and altered bowel habits. It can be subclassified in different subtypes according to the main clinical manifestation: constipation, diarrhea, mixed, and unclassified. Over the past decade, the role of gut microbiota in IBS has garnered significant attention in the scientific community. Emerging research spotlights the intricate involvement of microbiota dysbiosis in IBS pathogenesis. Studies have demonstrated reduced microbial diversity and stability and specific microbial alterations for each disease subgroup. Microbiota-targeted treatments, such as antibiotics, probiotics, prebiotics, synbiotics, fecal microbiota transplantation, and even diet, offer exciting prospects for managing IBS. However, definitive conclusions are hindered by the heterogeneity of these studies. Further research should focus on elucidating the mechanisms, developing microbiome-based diagnostics, and enabling personalized therapies tailored to an individual’s microbiome profile. This review takes a deep dive into the microscopic world inhabiting our guts, and its implications for IBS. Our aim is to elucidate the complex interplay between gut microbiota and each IBS subtype, exploring novel microbiota-targeted treatments and providing a comprehensive overview of the current state of knowledge.

## 1. Introduction

Irritable bowel syndrome (IBS) is a chronic functional gastrointestinal disorder characterized by abdominal pain and altered bowel habits, either in stool form or frequency, persisting for at least 6 months in the absence of an identifiable organic pathology [1].

According to the Rome IV criteria, IBS can be subclassified into four types, diarrhea-predominant IBS (IBS-D), constipation-predominant IBS (IBS-C), mixed IBS (IBS-M), and unclassified IBS (IBS-U), based on the main clinical presentation [2].

**Figure 1:**
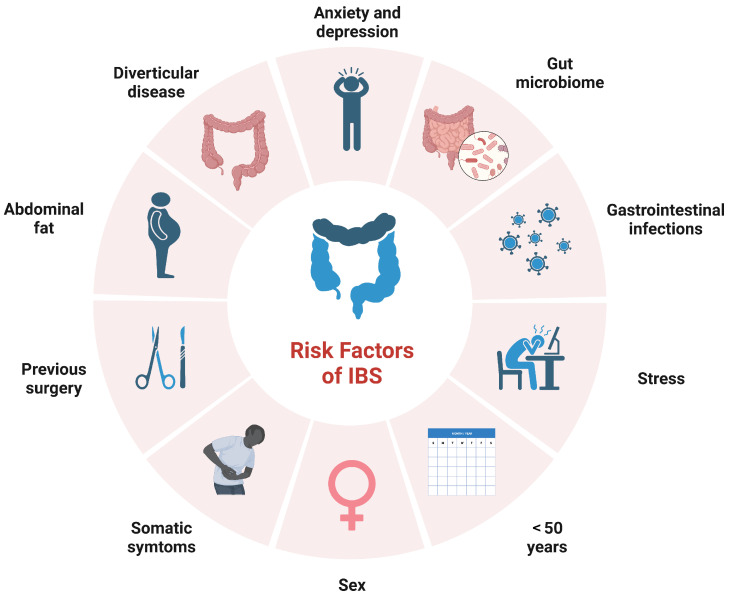
Main risk factors in irritable bowel syndrome (IBS).

It is challenging to ascertain precisely the global prevalence of IBS due to the lack of diagnostic biomarkers detectable in blood or stools, but current estimates suggest it affects approximately 5–10% of the population, in most countries [3]. The prevalence of IBS is higher in women compared to men [4]. It impacts individuals across all age groups, with symptom onset prior to 35 years for 50% of patients and a decreasing prevalence in patients over the age of 50 years [2]. Risk factors include a younger age, prior gastrointestinal infections, and stressful events, among others [5,6,7] (Figure 1). 

The pathophysiologic mechanisms underlying IBS remain unclear. Numerous pathogenic factors have been proposed, including alterations in gastrointestinal motility, visceral hypersensitivity, gut–brain axis dysfunction, low-grade intestinal inflammation and impaired epithelial barrier integrity [8,9,10]. IBS is often observed with other gastrointestinal and extraintestinal pathologies, as this syndrome has been connected especially to functional dyspepsia and gastroesophageal reflux disease, but also to fibromyalgia, chronic fatigue syndrome, chronic pain, and psychiatric conditions [11,12,13,14,15,16,17,18,19,20].

Emerging data suggest that gut microbiota dysbiosis may also contribute to disease development, even if plenty of evidence exhibited significant divergence due to heterogeneity, failing to delineate any uniform characteristic of the IBS-related microbiome, despite significant technological improvement over the past few years [11,21,22].

The commensal gut microbiota represents the largest symbiotic ecosystem in humans, exerting profound effects on health and disease, modulating biological processes [23]. It is also known to play a significant role in the pathogenesis of various gastroenterological diseases [24,25,26,27]. Growing evidence indicates a significant role for gut dysbiosis in IBS development, although specific microbial compositional changes and mechanisms remain to be fully elucidated [26]. Bidirectional relationships likely exist between dysbiosis and pathophysiological disturbances in IBS, and microbiome alterations may arise from and/or contribute to abnormalities in its pathogenesis [28].

This narrative review aims to provide a concise overview of the most recent evidence and developments regarding the role of the microbiota and its composition for the different IBS subtypes, comparing them to each other. We also explored the implications that these differences can bring to clinical practice by addressing microbiota-targeted treatment options for each disease subgroup.

## 2. Gut Dysbiosis in IBS

The gut microbiota comprises various types of intestinal micro-organisms including bacteria, archea, viruses, fungi and protozoa that coexist and perform functions such as dietary nutrient and drug metabolism, the maintenance of gut mucosal barrier integrity, immunomodulation, and pathogen protection [29]. Though only about one-third of bacterial species have been identified so far, the gastrointestinal tract is primarily composed of Firmicutes (64%), Bacteroidetes (23%), Proteobacteria (8%), and Actinobacteria (3%) [30]. 

An imbalance of gut flora can lead to dysbiosis which occurs via the loss or overgrowth of a particular organism, genetic mutations, or reduced microbial diversity, defined as the range of different micro-organisms present in the gut [31,32]. Microbial diversity has two main components—richness and evenness. Richness refers to the total number of different microbial species or taxa present in a community [33]. Evenness describes how evenly the microbes are distributed in terms of their relative abundances [33]. 

**Figure 2:**
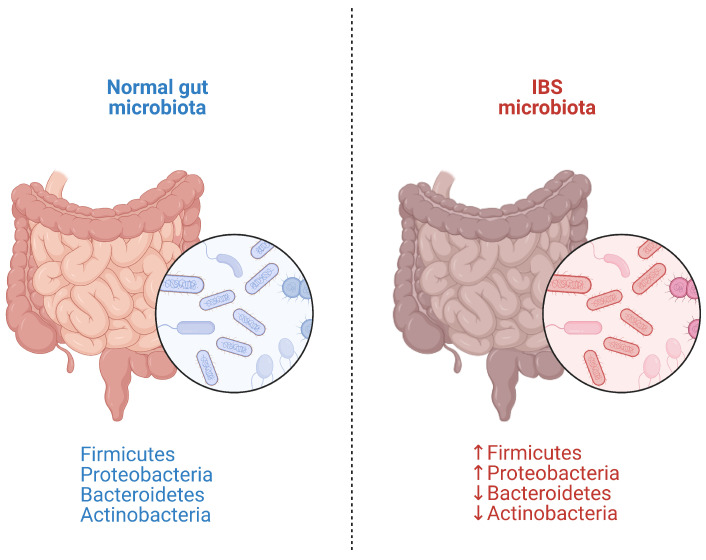
Main microflora alterations in irritable bowel syndrome (IBS).

Even slight disturbances in microbiota can cause inflammation, triggering oxidative stress, increased intestinal permeability, and, potentially, bacterial translocation across the mucosa [34]. Key differences have been found in the gut microbiota composition of IBS patients (Figure 2), although a signature microbiota has been associated with severe IBS, of which the intestinal microbiota characterization remains inconsistent with no accepted distinct trademark [35,36]. 

A systematic review evaluating 24 studies on microbiota changes in IBS patients showed a significant increase in *Enterobacteriaceae* (Proteobacteria phylum) and in *Bacteroides* (Bacteroidetes phylum), while *Faecalibacterium* (Firmicutes phylum) and *Bifidobacterium* (Actinobacteria phylum) were decreased compared to controls. However, the heterogeneity of the studies does not enable us to give consistent evidence of a specific IBS microbiota hallmark [37].

Likewise, a meta-analysis of 13 studies involving 360 IBS patients and 268 healthy individuals found reductions in *Bifidobacterium* (*p* < 0.001), *Lactobacillus* (*p* < 0.001), and *Faecalibacterium prausnitzii* (*p* < 0.001) in the IBS group [38]. Similar data were confirmed by a meta-analysis of 16 case-control studies, considering 777 IBS patients vs. 461 controls, which registered increased *Firmicutes* and decreased *Bacteroidetes* at phylum level [39]. An increase in *Clostridia* and *Clostridiales*, but a decrease in *Bacteroidia* and *Bacteroidales,* at lower taxonomic levels were also detected. Similarly, a meta-analysis of 23 studies on 1.340 subjects, evaluating the gut microflora in IBS patients vs. healthy individuals, found lower *Lactobacillus* (*p* < 0.01) and *Bifidobacterium* (*p* < 0.01), but higher *E. coli* (*p* < 0.01) and *Enterobacter* (*p* = 0.05) levels, without differences in *Enterococcus* or *Bacteroides* levels (*p* = 0.68 and 0.18, respectively) [40]. These findings highlight the need to delineate microbial signature changes specific to each IBS subtype in order to develop personalized therapeutic approaches targeting the dysbiosis associated with that subtype [41].

## 3. The Role of Dysbiosis in IBS Pathogenesis

Over the years, increasing interest has been given to the role of gut microbiota in the pathophysiology of IBS and its subtypes [42,43]. Nevertheless, the exact mechanisms and specific targets through which gut bacteria influence intestinal immunity have not yet been conclusively described [44].

Gut microbiota alterations, specifically, have been proven to play a relevant role in immune system activation and in intestinal barrier integrity [45]. This pathogenetic mechanism is particularly emphasized in the development of post-infectious IBS (PI-IBS), a condition where an acute infectious gastroenteritis or a systemic viral infection is followed by the emergence of IBS symptoms after 6–18 months of the acute episode, in patients without previous IBS clinical features [46]. In comparison to bacterial and viral enteritis, cases of protozoal infections pose a greater risk for the development of PI-IBS [47]. In this condition, a higher intestinal permeability and the exposure of the submucosa to foreign agents stimulating inflammation processes were described. Moreover, an incremented production of inflammatory cytokines and abdominal pain perception, known as visceral hypersensitivity, is observed [46,48,49,50,51]. For instance, according to one work of research, IBS-C patients showed a correlation between fecal *Lactobacillus* and *Bifidobacterium* with interleukin (IL)-10, whereas IBS-D patients showed a relationship between gram-positive and gram-negative bacteria and C-X-C motif chemokine ligand 11 [52].

Moreover, many studies demonstrated different fecal microbiota compositions in PI-IBS patients compared to non-PI-IBS patients and healthy controls, with evidence of reduced mucosal and fecal microbial diversity, lower levels of Clostridiales, and increased Bacteroides [53,54]. In addition, the analysis of the microbiota changes with qPCR assays comparing PI-IBS and IBS-D patients showed similar microbiota findings, suggesting a possible similar pathophysiology [54].

In the last three years, also, the influence of the SARS-CoV-2 infection on gut microbiota changes was investigated. For instance, an increase in Streptococcus, Enterococcus, and Corynebacterium species was observed in SARS-CoV-2-positive patients’ stool, while Faecalibacterium genus was decreased during an acute infection [55,56].

Emerging evidence suggests that molecular mimicry between bacterial and host proteins may offer a compelling mechanistic hypothesis for the development of IBS, particularly following infectious gastroenteritis or in combination with severe dysbiosis [57]. The translocation of bacteria past the disrupted epithelium and cross-reactive antibodies from bacteria–host epitope sharing may, together, induce persistent inflammation and nerve dysfunction, accounting for gastrointestinal symptoms [58]. In this context, an example is provided by cytolethal distending toxin B (CdtB), commonly produced by bacterial pathogens that cause gastroenteritis, such as *Campylobacter jejuni* [59]. Infection with *C. jejuni* causes post-infectious phenotypes in a rat model that are similar to IBS in humans [60]. The levels of host antibodies to CdtB have been correlated with small intestinal bacterial overgrowth (SIBO) [61]. Additionally, these antibodies can cross-react with the enteric neural protein vinculin, likely via molecular mimicry, potentially causing enteric nerve dysfunction and contributing to gut motor and sensory abnormalities [62].

Considering that only invasive bacteria can sustain high-titre IgG autoantibodies, identifying and replacing the bacteria that display cross-reactive epitopes can lead to strategies for restoring microbial balance and alleviating autoimmune-driven IBS symptoms [63].

The gut microbiota plays a significant role in the brain–gut microbiome axis and its influence on IBS. Communication between the gut microbiota and the central nervous system occurs through neuronal, endocrine, and immune signaling [64,65].

The enteric nervous system is involved in gastrointestinal function changes that impact microbiota composition [66]. For instance, patients with a higher abundance of *Prevotella*, compared to patients with an abundance *of Bacteroides,* showed notable changes in the right hippocampus, while data from another study reported a reduction in negative emotional responses in specific brain regions after the administration of certain probiotics like *Bifidobacterium longum* [67,68]. Metabolites produced by the gut microbiota, including short-chain fatty acids (SCFAs), serotonin, tryptophan, and tryptamine, play a significant role in regulating the gut-microbiome–brain axis [66,69,70]. SCFAs promote inflammatory cytokine production and the recruitment of immune cells, leading to neuroinflammation. Dysbiosis in IBS patients is associated with changes in the mucosal and systemic concentrations of microbial metabolites, further linking microbiota composition to psychological disorders [71]. The gut microbiota also affects the hypothalamic–pituitary–adrenal axis and influences stress-related dysbiosis and comorbid psychological conditions in IBS patients [65]. This bidirectional relationship influences the course of IBS, with a high prevalence of overlapping psycho-social comorbidity. Moreover, genetic factors seem to be involved in the development of IBS, suggesting a familial predisposition, from studies involving monozygotic twins and dizygotic twins [72]. Moreover, biomolecular epigenetic mechanisms can influence the pathogenesis of the disease through the dysregulation of the microbiota, which can lead to an increased production of metabolites such as sodium butyrate, a potent inhibitor of histone deacetylases [73,74]. The main interactions between microbiota and IBS pathogenetic mechanisms are summarized in Table 1.

## 4. Microbiota Changes in IBS Subtypes

### 4.1. Microbiota Composition in IBS Subtypes

Different studies have investigated changes in microbiota composition and their implications in different IBS subtypes (Table 2).

Specifically, one study with 16 patients and 21 controls reported a significant 1.2-fold lower diversity (*p* = 0.008) in IBS-D patients compared to healthy controls [76]. Regarding the microbial diversity, according to one study on fecal and mucosal samples from 33 IBS patients, mostly with IBS-D subtype (17/33), and 16 healthy controls, bacterial diversity was higher in fecal samples compared with mucosal ones (corrected *p* < 0.005) [77].

In contrast, another study reported a lower microbiota richness in the fecal samples of 27 IBS-D patients (*p* < 0.05), but no significantly lower diversity between these cases and healthy controls. Moreover, the major represented phyla in IBS-D microbiota were Bacteroidetes (64.6%), *Firmicutes* (26.1%), Fusobacteria (5.2%), and Proteobacteria (3.7%), albeit Bacteroidetes (56.4%), Firmicutes (35.9%), Proteobacteria (5.6%), and Fusobacteria (1.4%) were higher in the healthy subjects’ samples [78].

In terms of bacterial variety, the analysis of fecal microbiota of 23 IBS-D patients and 23 healthy controls resulted in significantly superior levels of the *Enterobacteriaceae* family (*p* = 0.03), and minor levels of the Faecalibacterium genera (*p* = 0.04) in the patient group compared to healthy subjects [79]. Moreover, one study conducted on 33 IBS-D patients and 15 healthy subjects analyzed the microbiota composition in different sites, such as duodenal and rectal mucosal samples. A significant difference between the duodenal and rectal microbiota in healthy subjects was reported (*p* = 0.003), while this difference was much lower in the IBS-D patients’ group (*p* = 0.052) [80]. Analyzing the fecal and small intestine mucosal microbiota of 37 IBS patients compared to 20 healthy subjects, *P. aeruginosa* was more frequently detected both in fecal samples (2.34% of total bacterial load) and small bowel samples (8.3% of total bacterial load) of patients with IBS compared to healthy subjects (*p* < 0.001) [81]. Another study involving 47 patients with IBS (20 with IBS-D, 20 with IBS-C, and 7 with IBS-U) analyzed fecal samples with quantitative polymerase chain reaction (qPCR), showing a significantly lower number of Lactobacillus in IBS-D patients than IBS-C patients (*p* = 0.002), while Bacteroides thetaiotamicron and segmented filamentous bacteria (from Bacillota phylum) were more numerous (corrected *p* = 0.001). Moreover, the species P. aeruginosa was more frequent in both IBS-D and IBS-C patients (97.9%), compared to healthy controls (33.3%; corrected *p* = 0.001) [82].

A systematic review and meta-analysis of 13 studies including 360 IBS patients and 268 healthy controls, only considering the qPCR analysis, compared the different IBS subtypes in terms of the microbiota. According to the presented data, a significant difference in IBS patients was reported for *Lactobacillus*, *Bifidobacterium,* and *Fecalibacterium prausnitzii* (*p* < 0.001), but not for *Bacteroides*-*Prevotella*, *Enterococcus*, *E. coli,* and *C. coccoides,* and this difference was especially driven by the IBS-D after subgroup analysis [83].

When comparing mucosal and fecal microbiota in IBS patient subgroups (13 IBS-D, 3 IBS-C, and 9 healthy subjects), a higher number of members of the *Enterobacteriaceae* and *Rikenellaceae* family were reported, respectively, in mucosal and fecal samples of IBS-C patients; more specifically, an increase of *Alistipes* and *Butyricimonas* were found in the first ones, while, in second ones, higher *Bacteroides* and lower *Coprococcus*, *Eubacterium*, *Fusobacterium*, *Haemophilus*, *Neisseria*, *Odoribacter*, *Streptococcus,* and *Veillonella* counts were registered. Instead, in the IBS-D subtype, there was an over-representation of *Acinetobacter*, *Butyricimonas*, *Leuconostoc,* and *Odoribacter* in feces and a reduction in *Desulfovibrio*, *Oribacterium*, *Brevundimonas,* and *Butyricicoccus* in the mucosal colonic samples [84]. In a larger study on 113 patients’ and 66 controls’ fecal samples, significantly lower butyrate-producing bacteria, such as Ruminococcaceae, unknown Clostridiales, Erysipelotrichaceae, and Methanobacteriaceae, were observed in IBS-D and IBS-M patients, when compared to the healthy group (corrected *p* = 0.002), while the microbiome of IBS-C patients did not show significant differences [85].

Concerning, more specifically, the IBS-C subtype, another research work analyzed through qPCR fecal samples of 62 patients with the IBS-D, IBS-C, and IBS-M subtypes compared to 46 healthy controls. The study reported significantly higher levels of Firmicutes, such as Clostridium species (*p* < 0.05), in IBS-C patients, and significantly reduced levels of Actinobacteria and Bacteroidetes phyla (*p* < 0.01) in this disease subtype [86].

Moreover, the analysis of rectal mucosa-associated microbiota of 27 IBS-D and 20 IBS-C patients compared to 26 healthy subjects showed higher levels of Bacteroidetes, Bifidobacterium, and *C. coccoides*/*E. rectale* (corrected *p* = 0.003), while bifidobacteria were lower in the IBS-D (corrected *p* = 0.011) group than in the IBS-C group and healthy controls [87]. From the analysis of the fecal microbiota of 14 IBS-C patients and 12 healthy subjects, several bacterial populations significantly differed between IBS-C patients and healthy controls [88]. The numbers of lactate-producing/utilizing bacteria and hydrogen-consuming microbes were at least 10× lower in IBS-C (*p* < 0.05). Conversely, sulphate-reducing bacteria utilizing lactate/hydrogen were 10–100× higher. The butyrate producer Roseburia-Eubacterium rectale was also lower in IBS-C (0.01 < *p* < 0.05). Fecal samples from IBS-C produced more sulphides and hydrogen, and less butyrate during starch fermentation than controls [88].

IBS-associated microbiota was also compared to some organic diseases. For instance, a research work evaluated IBS-D patients compared to ulcerative colitis (UC) patients. According to this trial involving 20 IBS-D patients, 28 UC patients (16 active and 12 inactive), and 16 healthy subjects, after a count of mucosa-associated microbiota using the fluorescent in situ hybridization (FISH) of mucosal biopsies, *E. coli*, Clostridium, and Bacteroides were significantly higher in IBS-D and UC patients, while Bifidobacteria were lower in UC and IBS patients compared to controls (*p* < 0.05) [89].

Other studies focused on the comparison between IBS, particularly IBS-D, and inflammatory bowel diseases [90,91,92]. Notably, in a model of human-microbiota-associated rats (HMAR) with induced experimental colitis, the IBS-C bacterial signature has shown anti-inflammatory properties with a reduction of pro-inflammatory cytokines [93]. 

Moreover, biofilms, a recently investigated entity recognized as an endoscopic finding in IBS and IBD patients, are considered a possible contributing factor in the IBS pathophysiology [94]. According to a study on 56 patients with IBS, 25 with IBD (specifically UC) compared to 36 healthy controls, biofilms are associated with a significant ten-fold elevation of primary bile acids in the intestinal lumen of IBS patients [95]. 

Even non-intestinal diseases were hypothesized to show similarities to IBS microbiota, specifically in a study which evaluated the microbiota changes in patients with IBS-D comparing to patients with depression by the analysis of fecal samples of 80 patients and 20 controls, reporting similar alterations in patients with IBS-D and depression, like higher Bacteroidetes and lower Firmicutes phylum levels [96].

In the context of IBS subtypes and clinical manifestations, the correlation between gut microbiota and symptoms severity was also investigated in several studies. In a research work on 110 IBS patients and 39 healthy controls, severe IBS patients exhibited lower microbial richness, a lower count of *Prevotella enterotype* and *Methanobacteriale*, and an increase in *Bacteroides* [36]. In another study with 80 IBS patients and 20 controls, IBS patients complaining of bloating had higher levels of *Ruminococcaceae* compared to patients without bloating (*p* < 0.05) [97]. Abdominal pain was also correlated with certain microbial taxa. Positive correlations were found with *Bacteroides* (*p* =  0.002), *Ruminococcus* (*p* = 0.004), and an unknown *Barnesiellaceae* (*p* = 0.041), while negative correlations were found with *Prevotella* (*p* = 0.003) and *Catenibacterium* (*p* = 0.019) [85]. Another research work reported that *Fecalibacterium*, reduced in the duodenal and rectal mucosal microbiota of 33 IBS-D patients compared to 15 healthy controls (4.1% vs. 1.8%, respectively), was negatively associated with abdominal bloating and stool consistency, while *Hyphomicrobium*, increased in the intestinal microbiota of these patients, was positively associated with abdominal pain and stool frequency [80].

### 4.2. Microbiota Metabolites in IBS Subtypes

Lately, attention has been given to the microbial-associated metabolites, which could represent possible biomarkers or targets for treatment, prospecting the opportunity to recognize in blood or stool samples specific disease patterns and therapeutic approaches, independently from the classifications already available, and then moving toward a personalized treatment [80]. 

In a study comparing IBS-D, IBS-C, and healthy controls (77 partecipants), fecal primary bile acids were significantly more represented in IBS patients, with an increase of unconjugated primary bile acids in IBS-D patients and lower SCFAs in the IBS-C group [74]. On the other hand, results from an additional study on 16 IBS-D, 15 IBS-C, and 15 healthy subjects showed an increase in primary bile acids and a decrease in secondary bile acids in IBS-D patients’ stool samples [98]. Similar but not significant differences were observed in IBS-C patients, with evidence of a significantly lower deconjugation activity in both IBS-D (*p* = 0.0001) and IBS-C patients (*p* = 0.005) compared to healthy controls [98]. Another analysis conducted on 14 patients with IBS-D, besides the evidence of higher levels of primary bile acids (*p* = 0.02) and lower secondary bile acids (*p* = 0.03), reported a significant correlation with clinical symptoms such as stool frequency and consistency compared to healthy subjects [99]. Another larger study including 345 IBS-D patients evidenced that the increase of *Clostridia* was associated with the rise of fecal bile acids and with a decrease in serum fibroblast growth factor 19 (FGF19) concentration, a feedback regulator of intestinal transit, possibly explaining the augmented bowel frequency and stool water content in this IBS subtype. If confirmed, this could represent a possible biomarker in IBS-D [100]. Based on a systematic review and meta-analysis of 15 studies (case-control studies, RCTs, and self-controlled studies) on the variations of fecal SCFAs in 448 IBS patients, there was a significant increase of fecal propionate in patients compared to healthy controls; particularly, there were lower levels of propionate (standard mean difference, SMD = −0.91) and butyrate (SMD = −0.53) in patients with IBS-C, while the concentration of butyrate was higher in IBS-D patients (SMD = 0.34) than in healthy subjects [101]. 

According to emerging evidence, other gut microbiota metabolites like amino acids, neurotransmitters, and vitamins can play a role in the pathophysiology of IBS and could be targets for treatment [102].

### 4.3. Methanogenic Species in IBS Subtypes

Gut microbiota is also involved in the production of various gases through the fermentation of carbohydrates, including carbon dioxide, hydrogen, and methane, which results in the highest prevalence detected. The intestinal tract harbours not only bacteria but also archaea, such as methanogenic species. *Methanobrevibacter smithii*, *Methanobrevibacter oralis*, *Methanobacterium ruminantium,* and *Methanosphaera stadtmania* are dominant archaeal and methane-producing species in the human gut [103,104]. Animal studies showed methane slows transit through the small intestine, potentially contributing to constipation [105,106]. A prospective, double-blind study directly comparing methane positivity on lactulose breath tests (LBTs) to the Rome I IBS classification found methane positivity had a sensitivity of 92% and specificity of 81% for correctly identifying patients with IBS-C [107]. Additionally, a meta-analysis of nine other studies including 1.277 patients demonstrated a positive association between elevated methane levels and IBS-C diagnosis [108]. Concordantly, a reduction of methane-producing micro-organisms was observed in IBS-D and IBS-M [85]. Studies have also demonstrated methane quantitatively influences the degree of constipation in LBS patients. A direct proportional relationship was observed between quantitative methane levels at LBTs and the severity of patient-reported constipation symptoms, including decreased stool frequency and harder stool consistency [109]. Additionally, methanogenesis correlated positively with the severity of bloating and flatulence (*p* < 0.01) as higher gas volumes induce more distension [110]. As a potential therapeutic option, a lactone form of lovastatin was observed to inhibit enzymes involved in the methanogenic pathway and reduce symptoms associated with this subclass of IBS [111].

SIBO is a condition characterized by abnormally high concentrations of bacteria in the small intestine, presenting with abdominal distension, bloating, and diarrhea, as main symptoms [112,113]. In clinical practice, to diagnosis this condition, culture methods have been replaced by non-invasive breath tests [114].

A meta-analysis of 25 studies including 3.192 IBS subjects and 3.320 controls found the prevalence of SIBO in IBS was 31% (95% CI 29.4–32.6; OR = 3.7, 95% CI 2.3–6.0, *p* = 0.001) compared to controls (16%) [115]. More specifically, to associate SIBO and archaeal methanogenesis, the American College of Gastroenterology’s clinical practice guideline introduced the concept of intestinal methanogen overgrowth (IMO) [116]. A systematic review including 17 studies and 1653 patients evaluated the relationship between methane-positive SIBO and IBS. Data showed a prevalence of 25% (95% CI 18.8–32.4); methane production was not significantly increased in overall IBS patients compared to controls (OR = 1.2, 95% CI 0.8–1.7, *p* = 0.37), but it was significantly more prevalent in IBS-C compared to IBS-D (OR = 3.1, 95% CI 1.7–5.6, *p* = 0.0001) [117]. On the other hand, a meta-analysis found the association between SIBO and IBS subtypes was strongest for IBS-D, with a prevalence of 35.5% (95% CI 32.7–40.3), or 25.2% (95% CI 22.2–28.4) for IBS-M, compared to 22.5% (95% CI 18.1–26.9) for IBS-C [118]. To fully comprehend the role of SIBO and methanogenic archea, new evidence that clarifies the mechanisms that interact between the parties involved are required.

**Table 2:** Selected literature review about microbiota alterations and IBS subgroup.

Author (Year)	Country	Study Type	Sample Size	IBS Subtype	Type of Analysis	Sample Type	Rome Criteria	Results
Carroll et al. (2011) [77]	USA	Prospective monocentric	37 (16/21)	IBS-D	16S rRNA T-RFLP	Fecal Mucosal	Rome III	Higher level of microbial biodiversity in fecal- than in mucosal-associated communities within IBS-D (*p* = 0.008)
Rajilić-Stojanović et al (2011) [87]	Europe	Prospective monocentric	108 (62/46)	IBS-D IBS-C IBS-M	16S rRNA qPCR	Fecal	Rome II	IBS-C had increased Firmicutes (including *Clostridium* spp.) (*p* < 0.05) and decreased Actinobacteria and Bacteroidetes (*p* < 0.01) vs. controls
Durbàn et al. (2012) [85]	Europe	Prospective monocentric	16/9	IBS-D IBS-C	16S rRNA qPCR	Fecal Mucosal	Rome II	IBS-D had increased fecal *Acinetobacter* (OR = 16.7, *p* = 0.02), *Butyricimonas* (OR = 2.29, *p* = 0.004), and *Odoribacter* (OR = 6.11, *p* = 0.003), but decreased mucosal Oribacterium (OR = 0.17, *p* = 0.04), *Brevundimonas* (OR = 0.09, *p* = 0.0009), and Butyricicoccus (OR = 0.38, *p* = 0.026) vs. controls. IBS-C had increased fecal Alistipes (OR = 5.82, *p* = 0.01) and *Butyricimonas* (OR = 3.27, *p* = 0.004) and increased mucosal Bacteroides (OR = 3.15, *p* = 0.003), but decreased *Coprococcus* (OR = 0.03, *p* = 0.007), Fusobacterium (OR = 0.02, *p* = 0.003), Streptococcus (OR = 0.06, *p* = 0.007), and *Veillonella* (OR = 0.03, *p* = 0.04) in feces vs. controls.
Carroll et al. (2012) [80]	USA	Prospective monocentric	46 23/23	IBS-D	16S rRNA qPCR	Fecal	Rome III	Higher Enterobacteriaceae (*p* = 0.03) and lower Fecalibacterium genera (*p* = 0.04)
Parkes et al. (2012) [88]	Europe	Prospective monocentric	47/26	IBS-D IBS-C	FISH	Mucosal (rectum)	Rome III	IBS-D had lower bifidobacteria vs. IBS-C and controls (*p* = 0.011). In IBS, maximum stools/day negatively correlated with mucosal Bifidobacteria (*p* < 0.001) and Lactobacilli (*p* = 0.002).
Chassard et al. (2012) [89]	Europe	Prospective multicentric	14/12	IBS-C	16S rRNA FISH	Fecal	Rome II	IBS-C had lower lactate-producing/utilizing bacteria and methanogens/acetogens (*p* < 0.05), but 10–100× higher H2/lactate-utilizing sulfate reducers vs. controls. Roseburia-*E. rectale* butyrate producers were lower in IBS-C (*p* < 0.05–0.01). Mucosal vs. fecal microbiota differed significantly (*p* = 0.002) regardless of IBS characteristics. Mucosal microbiota was dominated by Bacteroidetes, fecal by Firmicutes/Actinobacteria/Proteobacteria. Controls had higher uncultured Clostridiales (*p* < 0.005) in mucosa than IBS.
Rangel et al. (2015) [78]	Europe	Retrospective monocentric	49 33/16	IBS-D IBS-C IBS-M IBS-U	16S rRNA Phylogenetic microarray	Fecal Mucosal	Rome III	Mucosal vs. fecal microbiota differed significantly (*p* = 0.002), independent of IBS characteristics. Mucosa was dominated by Bacteroidetes, feces by Firmicutes/Actinobacteria/Proteobacteria. Controls had higher uncultured Clostridiales (*p* < 0.005) in mucosa vs. IBS. Fecal bacterial diversity higher than mucosal in IBS (*p* < 0.005).
Pozuelo et al. (2015) [86]	Europe	Prospective multicentric	113/66	IBS-D IBS-C IBS-M	16S rRNA qPCR	Fecal	Rome III	IBS had lower microbial diversity associated with lower butyrate-producing bacteria, especially in IBS-D/M (*p* = 0.002). Untreated IBS had lower Methanobacteria vs. controls (*p* = 0.005). Bacterial taxa was correlated with flatulence/abdominal pain (*p* < 0.05).
Shukla et al. (2015) [83]	Asia	Prospective monocentric	47/30	IBS-D IBS-C IBS-U	16S rRNA qPCR	Fecal	Rome III	Relative difference of Bifidobacterium (*p* = 0.042) was lower, while Ruminococcus productus-Clostridium coccoides (*p* = 0.016), *Veillonella* (*p* = 0.008), Bacteroides thetaiotamicron (*p* < 0.001), and *Pseudomonas aeruginosa* (*p* < 0.001) were higher among IBS patients than controls. Lactobacillus (*p* = 0.002) was lower, while *Bacteroides thetaiotamicron* (*p* < 0.001) and segmented filamentous bacteria (SFB, *p* < 0.001) were higher among IBS-D than IBS-C. Numbers of Bacteroides thetaiotamicron (*p* < 0.001), *P. aeruginosa* (*p* < 0.001), and Gram- (*p* < 0.01) were higher among IBS-C and IBS-D than controls. Quantity of SFB was higher among IBS-D (*p* = 0.011) and lower among IBS-C (*p* = 0.002). *Veillonella* species was higher among IBS-C than controls (*p* = 0.002).
Liu et al. (2017) [84]	Europe Asia USA	Systematic review and meta-analysis	360 (13 studies)	IBS-D IBS-C IBS-M	qPCR	Fecal Mucosal	Rome II Rome III	Subgroup analysis showed IBS-D patients had significantly different expression of Lactobacillus (SMD = −1.81, *p* < 0.001) and Bifidobacterium (SMD = −1.45, *p* < 0.001).
Zhuang et al. (2018) [79]	Asia	Prospective monocentric	43 30/13		16S rRNA pyrosequencing	Fecal	Rome III	IBS-D had decreased fecal microbiota richness (*p* < 0.05) but not diversity vs. controls: Bacteroidetes (64.6%), Firmicutes (26.1%), Fusobacteria (5.2%), and Proteobacteria (3.7%).
Li et al. (2018) [81]	Asia	Prospective Monocentric	33/15	IBS-D	16S rRNA pyrosequencing	Mucosal (duodenum + rectum)	Rome III	Mucosal microbiota in duodenal samples differed from rectal samples in HC (*p* = 0.003), while less difference was shown in IBS-D (*p* = 0.052). Identified 24 genera were shared in duodenum and rectum, which both changed in IBS-D.
Sun et al. (2019) [102]	Europe, USA, Asia, Australia	Systematic review and meta-analysis	448 (15 studies)	IBS-D IBS-C IBS-M IBS-U	16S rRNA	Fecal	Rome I Rome II Rome III	IBS had higher fecal SCFAs vs. controls (SMD = 0.44). IBS-C had lower propionate (SMD = −0.91) and butyrate (SMD = −0.53) than controls. IBS-D had higher butyrate than controls.
Wang et al. (2020) [41]	Europe USA Asia	Systematic review and meta-analysis	208	IBS-D	16S rRNA qPCR	Fecal	Rome IV	Lower Lactobacillus (MD = −0.62 log10CFU/g); Lower Bifidobacterium (MD = −0.86 log10CFU/g); Higher *E. coli* (MD = −40.77 log10CFU/g); Lower Lactobacillus (MD = −0.43 log10CFU/g); Lower Bifidobacterium (MD = −1.76 log10CFU/g).
			105	IBS-C		Fecal		

IBS-D = irritable bowel syndrome—diarrhea; IBS-C = irritable bowel syndrome—constipation; IBS-M = irritable bowel syndrome—mixed; rRNA = ribosomal ribonucleic acid; T-RFLP = terminal-restriction fragment length polymorphism; qPCR = quantitative polymerase chain reaction; OR = odds ratio; CFU = colony-forming units, MD = mean deviation; FISH = fluorescence in situ hybridization.

Overall, one of the limitations of many studies is the use of diagnostic criteria that do not always correspond to those of Rome IV, also for chronological reasons. Another missing point concerns the absence of a long-term follow-up in several investigations. Moreover, the lack of standardization in the type of sample to be analyzed for microbiota analysis inevitably creates notable heterogeneity. 

## 5. Microbiota-Targeted Therapeutic Approaches in IBS

Concerning the therapeutic application of microbiota in functional intestinal disorders (Table 3), many studies have been conducted, but strong evidence of a clinical implementation is accepted only for the diarrhea-predominant subtype.

### 5.1. Antibiotics

As mounting evidence shows intestinal dysbiosis contributes to IBS pathogenesis, antibiotics are emerging as a potential therapeutic approach. The most studied one for IBS treatment is rifaximin, a broad-spectrum oral antibiotic with minimal systemic absorption, a favourable side effect profile, and low risk of resistance [93]. Several studies have examined the effect of rifaximin on the gut microbiota of patients with IBS, searching for an association between its clinical manifestation and the intestinal microenvironment [119]. A meta-analysis of five randomized controlled trials (RCTs) comparing rifaximin to a placebo for IBS found rifaximin yielded statistically significant IBS symptom improvement (symptoms persisting RR = 0.84; 95% CI 0.79–0.90) [119]. Compared to other antibiotics like neomycin, ciprofloxacin, doxycycline, and amoxicillin/clavulanate, rifaximin demonstrated greater efficacy and lower resistance concerns in IBS patients (*p* < 0.01) [120]. Rifaximin is principally used in the IBS-D subtype, demonstrating efficacy and safety in many different studies. Two large identically designed multicentre RCTs (TARGET 1 and 2) administered rifaximin (550 mg three times daily) or a placebo for 14 days to IBS-D patients [121]. Significantly, the rifaximin group showed overall IBS symptom improvement one month post-treatment compared to placebo (40.7% vs. 31.7%, *p* < 0.001, in the two studies combined). One study assessed the effect of rifaximin assumption (400 mg twice a day) for two weeks in IBS-D patients, showing no significant effect in fecal microbiota richness and diversity before and after antibiotical administration, even if some variations in microbial composition after treatment were detected, defining some potential biomarker bacteria of IBS-D. However, rifaximin treatment showed a significant reduction of clinical symptoms, such as abdominal pain, urgency, diarrhea, abdominal distension, and discomfort in the treatment group after 10 weeks [86]. In addition, the efficacy of rifaximin on clinical manifestation (*p* < 0.05) was confirmed in patients with IBS-D, independently from the presence of concomitant SIBO [122]. Another RCT observed, after a two-week rifaximin course (550 mg three times daily), a significantly lower relative abundance of some taxa like Peptostreptococcaceae, Verrucomicrobiaceae, and Enterobacteriaceae, which was, in any case, transient to the end follow-up analysis [123]. Conversely, after 2 weeks of rifaximin treatment, an increase in the fecal Bifidobacterium, and a reduction of E. coli and Enterobacterbesides was observed in IBS-D patients with a different baseline microbial composition from the healthy controls [124]. Due to this pool of evidence, the American College of Gastroenterology, in its latest guidelines, recommends rifaximin for IBS-D-predominant treatment [125]. Several studies reveal changes in microbiota species richness and the relative abundance of select bacteria following rifaximin treatment in IBS patients, although the changes may be transient [123]. Further research is needed to better characterize rifaximin’s effects on the microbiome. Overall, current evidence supports antibiotics like rifaximin as an emerging IBS treatment targeting intestinal dysbiosis, but additional investigation is warranted.

### 5.2. Probiotics, Prebiotics, Synbiotics, and Postbiotics

Probiotics are live micro-organisms orally administered that can colonize the gastrointestinal tract [126]. Prebiotics, on the other hand, are non-digestible food components that provide benefits to the host by selectively promoting the growth and/or activity of health-promoting bacteria [127]. Synbiotics, instead, are a mix of both probiotics and prebiotics [128]. Lastly, postbiotics, or biogenics or metabiotics, are a group of bioactive compounds that are produced by bacteria, including bacteriocins, vitamins, and SCFAs, that play a beneficial role for the host [129].

In recent years, research has been conducted investigating the potential of these components for the management of IBS [130]. Studies have largely demonstrated the positive effects of probiotics on IBS, although therapeutic efficacy appears to be species-specific. A randomized control trial (RCT) reported that, compared to *Lactobacillus salivarius UCC4331*, *Bifidobacterium infantis* 35624 significantly normalized the abnormal interleukin 10/12 ratio and alleviated all symptoms with the exception of bowel movement frequency and consistency [131]. In addition, the beneficial effects of *B. infantis* 35624 appeared to be dose-dependent, suggesting the probiotic species and dose are important determinants of therapeutic efficacy. Several RCTs demonstrated that treatment with specific probiotics, like *Lactiplantibacillus plantarum*, *Bacillus coagulans,* or multi-strain probiotic formulations, results in a significant improvement in the abdominal pain, bowel frequency, and quality of life in patients with IBS-D and, in some cases, a better response in patients with a specific microbial composition at baseline [132,133,134,135]. In contrast, other evidence suggested a microbial composition modification after probiotic treatment, with the effect of a clinical improvement on IBS-affected patients, but the beneficial efficacy did not depend on the probiotic-induced microbial changes [136]. Multiple RCTs studied the effects of *S. cerevisiae* supplementation in IBS patients. Overall, *S. cerevisiae* administration led to a significant response to abdominal pain and stool consistency in patients with IBS of all subtypes [137]. According to another RCT, the main effect was observed in the first month of treatment, with no statistical difference in bowel frequency and on the stool Bristol scale, but with a significant improvement in the quality-of-life score after 2 months of therapy [138]. Other trials reported no significant clinical effects of *S. cerevisiae* in IBS treatment, but also noticed that the main beneficial effects on symptoms were imputable to the IBS-C population [139,140,141]. A systematic review and meta-analysis showed that some strain-specific probiotics are associated with improvement in abdominal pain in IBS, notably *B. coagulans MTCC5260* (RR = 4.9, 95% C.I. 3.3, 7.3), *S. boulardii CNCM I-745* (RR = 1.5, 95% C.I. 1.1, 2.1), *L. plantarum 299v* (RR = 4.6, 95% CI 1.9, 11.0), and *S. cerevisiae CNCM I-3856* (RR = 1.3, 95% C.I. 1.04, 1.6) [142]. Based on another systematic review and meta-analysis on the administration of probiotics in IBS-D patients, it emerged that their assumption resulted in a significant improvement of the symptom score, abdominal pain, and abdominal distension (*p* < 0.05), but did not significantly modified the patients‘ quality of life [143]. A better efficacy of double-coated probiotics versus non-coated probiotics in the treatment of IBS-D was also suggested [144]. Moreover, concerning the IBS-C subtype, it was observed that the intake of *L. helveticus* for a week relieved abdominal symptom associated with constipation and reduced the intestinal transit time, while adding polydextrose, a synthetic soluble fiber polymer, did not show a significant improvement [145]. A meta-analysis of 10 studies, involving a total of 757 patients, evaluated the role of probiotics for IBS-C and found significantly improved stool consistency and increased fecal *Bifidobacterium* and *Lactobacillus* levels compared to the placebo [146]. However, no significant differences were observed in abdominal pain, bloating, quality of life, or adverse events. 

Despite the small number of high-quality studies, the results suggest probiotics may benefit IBS-C patients by improving stool consistency and gut microbiota composition, with a favourable safety profile. Additional robust RCTs are warranted to confirm probiotic efficacy and optimal strains for IBS-C.

The role of prebiotics in IBS has extensively been evaluated, with dietary carbohydrates and more specific inulin-type fructans and galactooligosaccharides being the most extensively studied [147,148]. A meta-analysis examined 11 RCTs involving 729 patients, investigating prebiotics for IBS, finding no differences between the prebiotic and placebo groups in response rates, abdominal pain, bloating, or quality of life [149]. However, flatulence severity was improved at doses ≤6 g/d or with non-inulin fructans, while inulin worsened it. Prebiotics also seemed to elevate Bifidobacteria abundance, though a risk of bias was present across studies [149]. Some evidence highlighted the possible role of prebiotics such as partially hydrolyzed guar gum for the management of IBS-D-like symptoms, leading also to an increase of Bifidobacterium (*p* < 0.05) [150,151]. Differently, the role of soluble fibers is well-recognized in the IBS-C subgroup as having the potential to improve quality of life and regulate colonic transit, in contrast to insoluble fiber intake which increases colonic volume and may exacerbate bloating [152,153].

Literature examining synbiotics for IBS showed mixed results. Some synbiotics with probiotics like *Lactobacillus* and *Bifidobacterium,* in addition to prebiotics like fructooligosaccharides, failed to improve IBS symptoms in multiple studies [154,155,156], while other studies showed benefits. Moreover, certain synbiotics reduced flatulence, abdominal pain, diarrhea, and bloating, but efficacy appears to depend on the specific probiotic strains and prebiotics used [157,158]. 

Postbiotics recently emerged as a potential IBS treatment, with preliminary evidence showing promising effects on the inflammatory and immune pathways in an ex vivo model of post-infectious IBS [159]. Moreover, the possible effectiveness of microencapsulated sodium butyrate supplementation was investigated, with a significant improvement of abdominal pain severity, flatulence, diarrhea, constipation, urgent bowel movements, nausea, and vomiting (*p* < 0.001) after 3 months’ assumption. Another study evaluated short-chain fructooligosaccharides (scFOSs), demonstrating it to be effective on rectal sensitivity, microbiota composition, symptom recovery, and quality of life improvement in almost 80 IBS patients. The effect of this treatment was more accentuated in the IBS-C subtype (*p* = 0.051). Furthermore, scFOSs determined a significant reduction of abdominal distension and significant growth in fecal *Bifidobacteria*, but not in the other bacterial groups [160]. 

Limited and heterogeneous data from existing prebiotic, probiotic, symbiotic, and postbiotic studies prevented the widespread acceptance for IBS treatment. The American Gastroenterological Association currently has no recommendations supporting probiotics for IBS, while the American College of Gastroenterology recommends against the use of probiotics, given the very low quality of evidence for efficacy [125]. Further large-scale RCTs are required in order to clarify the role of these compounds in IBS therapy.

### 5.3. Fecal Microbiota Transplantation

Fecal microbiota transplantation (FMT), already largely used in the treatment of *Clostridium difficile* recurrent infection since its approbation by the FDA in 2013, was also investigated in patients with IBS, yielding conflicting results [161,162,163,164]. In a double-blind placebo-controlled RCT of 165 patients who received 30 g FMT and 60 g FMT vs. placebo (own feces) in a 1:1:1 ratio, a significant improvement of symptoms 3 months post-FMT in both experimental groups compared to placebo was registered [165]. In another double-blind placebo-controlled RCT, researchers assigned 90 patients (2:1) to receive a colonoscopy FMT vs. placebo (own feces), finding a significant improvement in IBS symptoms based on IBS Severity Scoring System scores (IBS-SSS) post-FMT in the experimental group [166]. Similarly, in another double-blind RCT, patients with refractory IBS who underwent a nasojejunal administration of donor stool experienced a significant improvement in IBS-related symptoms compared to the placebo group who received autologous stool [167]. A systematic review and meta-analysis of nine RCTs confirmed that FMT significantly improved the symptoms and QoL compared to placebo in patients with IBS syndrome. Improvements were seen in symptom severity scores, clinical response rates, and quality of life scores up to 3 years after a single transplantation (RR = 2.5, 95% CI 1.6, 3.7) [168]. On the other hand, in a double-blind placebo-controlled RCT of 52 participants who received FMT vs. placebo capsules for 12 days, investigators found a significant reduction in the IBS-SSS and quality of life scores after 3 months in patients who received the placebo [169]. In another double-blind RCT, FMT administered in capsule form for 12 days did not significantly improve abdominal pain, stool frequency, or stool form in patients with moderate-to-severe IBS during treatment or at the 1-, 3-, or 6-month follow-up; however, a statistically significant improvement in stool frequency in the FMT group was registered when examining the improvement in stool frequency during treatment compared to post-treatment and one month post-treatment [170]. Similarly, a meta-analysis which pooled data from 254 participants across four studies found no significant improvement in IBS symptoms in patients who received FMT vs. placebo after 12 weeks (RR = 0.93; 95% CI 0.48–1.79) [171]. Another meta-analysis which contained data from five RCTs and 267 patients found that IBS symptoms did not significantly improve post-FMT (RR = 0.98; 95% CI 0.58–1.66) regardless of stool type, but some administration methods yielded a better result than others [172]. Additionally, an observational RCT specifically examined the therapeutic efficacy of FMT for IBS-D patients with anxiety and depression versus oral placebo capsules. The experimental group showed an IBS-SSS score reduction, improved stool consistency, and decreased anxiety and depression scores after 12 weeks of treatment, also increasing the bacterial alpha diversity and the relative abundance of *Bacteroidetes* and *Firmicutes* compared to control (50.6% vs. 47.6% and 45.5% vs. 38.9%, respectively) [173]. Given the substantial conflicting data, further investigation is needed to better characterize the potential role of FMT in the treatment of IBS.

### 5.4. Diet

Over the past decade, dietary modifications have increasingly emerged as a valid treatment approach for IBS [174]. Dietary advice for IBS draws from guidelines including the National Institute for Health and Care Excellence (NICE) [175] and British Dietetic Association (BDA) [176], underlining self-management through lifestyle, diet, exercise, and symptom-based medication education to empower patient control via multifactorial trigger optimization. Recently, emphasis has centered on the low-FODMAP (fermentable oligosaccharides, disaccharides, monosaccharides, and polyols) diet, which demonstrated efficacy in alleviating certain clinical manifestations when compared to BDA/NICE dietary advice (specifically bloating and abdominal distension; RR = 0.72; 95% CI 0.55–0.94) for IBS patients based on the accumulated research evidence [177]. Many individuals are sensitive to FODMAPs, which can increase symptoms such as bloating, diarrhea, gas, constipation, or abdominal pain mimicking IBS. If malabsorbed, FODMAPs exert a potent osmotic effect causing water influx into the colon, resulting in diarrhea, or they may be fermented by colonic bacteria, inducing excessive gas production. Visceral hypersensitivity, often seen in IBS subjects, could exacerbate after intestinal distension triggered by gas or fluids, hence aggravating IBS abdominal sensations [178]. In theory, lessened FODMAP consumption diminishes fluid retention in the gut and relieve symptoms, but a low-FODMAP diet also decreases fiber intake, possibly inducing constipation in some patients [179]. While dietary interventions are important for managing IBS symptoms, approximately one-third of patients still report inadequate relief when adhering to evidence-based dietary advice [180]. 

Several studies, both prospective controlled and uncontrolled trials, evaluated the efficacy of the low-FODMAP diet in patients with IBS, demonstrating promising results in the clinical practice, even though heterogeneous findings were reported. A comprehensive systematic review and meta-analysis on 12 trials including 772 patients investigated the effects of a low-FODMAP diet on IBS symptoms, showing a significant improvement of IBS severity, according to the IBS Severity Scoring System (IBS-SSS), in most studies and higher IBS-QoL scores in patients with the low-FODMAP diet versus controls [181]. Many studies have investigated the effect of diet in IBS patients and gut microbiota modifications related to some specific diet regimens and especially the effects of a low-FODMAP diet on the colonic microbiota composition [182,183,184]. A low-FODMAP diet was found to alter the composition of the gut microbiota by significantly reducing the levels of Bifidobacteria and decreasing the overall bacterial count [185,186]. According to a recent systematic review and meta-analysis on the effects of a low-FODMAP diet on the colonic microbiome in IBS patients, evaluating nine trials on 403 patients, no definitive impacts were observed on the diversity of the gut microbiome, fecal concentrations of specific SCFAs, or fecal pH [187]. Recent research indicates an individual’s response to a low-FODMAP diet may hinge upon their gut microbiota composition, although no specific microbial signature has been delineated that reliably predicts a positive outcome [188,189]. 

Regarding the impact of a low-FODMAP diet among distinct IBS subtypes, current evidence presents an inconclusive picture. Regarding the IBS-D subtype, the low-FODMAP diet has become a widely suggested dietary approach for dealing with abdominal pain and bloating, without unequivocal results on improved stool composition [190,191], whereas, in the IBS-C subtype, according to a systematic review on dietary modifications in patients with chronic constipation and IBS-C, poor evidence was observed in the examined studies, due to heterogeneity in subject selection, treatment, and outcome assessment [192].

In addition, the relationship between routine FODMAP consumption and symptom severity was evaluated in 189 IBS patients’s (54 IBS-D, 46 IBS M, 46 IBS-U, 44 IBS-C) recorded nutritional intake over four days [193]. Symptom severity evaluated through IBS-SSS revealed small differences in FODMAP intake across IBS subtypes, with dietary effects varying depending on subtype. Distinct FODMAP components seemingly participate differently in symptom generation, necessitating the evaluation of each FODMAP separately in the IBS subtypes using randomized controlled trials or longitudinal studies [194].

**Table 3:** Selected literature review about microbiota-targeted therapeutic approaches in IBS.

Author (Year)	Therapy	Study Type	Sample Size	Results	IBS Subtype
**ANTIBIOTICS**					
Pimentel et al. (2011) [123]	Rifaximin	Two identically designed, phase 3, double-blind, placebo-controlled RCTs	1260	More patients in rifaximin group had relief from global IBS symptoms during the first 4 weeks after treatment vs. placebo (40.7% vs. 31.7%, *p* < 0.001). More rifaximin patients had adequate bloating relief versus placebo (40.2% vs. 30.3%, *p* < 0.001,). Rifaximin patients responded with daily IBS symptom, bloating, abdominal pain, and stool consistency ratings.	IBS-D
Lembo et al. (2016) [122]	Rifaximin	Phase 3, randomized, double-blind, placebo-controlled trial	1074	Rifaximin had a greater percentage of responders than placebo (38.1% vs. 31.5%, *p* = 0.03), specifically for abdominal pain (50.6% vs. 42.2%, *p* = 0.018) but not stool consistency (51.8% vs. 50.0%, *p* = 0.42). Rifaximin also showed significant improvements in recurrence prevention, durable response, and bowel urgency compared to placebo. Adverse events were low and similar between groups.	IBS-D
Fodor et al. (2019) [124]	Rifaximin	Phase 3, randomized, double-blind, placebo-controlled trial	103	Rifaximin treatment resulted in significantly lower relative abundance of seven taxa such as Peptostreptococcaceae, Verrucomicrobiaceae, and Enterobacteriaceae (10% false discovery rate). However, these effects were short-term, with no significantly different changes in taxa abundance at the end of the 46-week study versus baseline.	IBS-D
**PROBIOTICS, PREBIOTICS, SYNBIOTICS, POSTBIOTICS**					
Wang et al. (2022) [144]	Probiotics	Systematic review and meta-analysis	943 (10 RCTs)	Significantly decreased of IBS-D symptom score (SMD = −0.55, 95% CI: −0.83, −0.27, *p* < 0.05), abdominal pain (SMD = −0.43, 95% CI: −0.57, −0.29, *p* < 0.05), and abdominal distension (SMD = −0.45, 95% CI: −0.81, −0.09, *p* < 0.05) compared to placebo.	IBS-D
Shang et al. (2022) [147]	Probiotics	Systematic review and meta-analysis	757 (10 RCTs)	Probiotics significantly improved stool consistency (SMD = 0.72, 95% CI (0.18–1.26), *p* < 0.05, low quality) and increased the number of fecal Bifidobacteria (SMD = 1.75, 95% CI (1.51–2.00), *p* < 0.05, low quality) and Lactobacillus (SMD = 1.69, 95% CI 1.48–1.89, *p* < 0.05, low quality), while no significant differences were found in abdominal pain scores, bloating scores, QoL scores, or the incidence of adverse events (*p* >0.05)	IBS-C
Wilson et al. (2019) [150]	Prebiotics	Systematic review and meta-analysis	729 (11 RCTs)	No difference between groups (OR = 0.62; 95% CI: 0.07–5.69; *p* = 0.67). No differences found for severity of abdominal pain, bloating, and flatulence, and QoL score between prebiotics and placebo. However, flatulence severity was improved by prebiotics at doses ≤6 g/d (SMD: –0.35; 95% CI: –0.71–0.00; *p* = 0.05) and by non-inulin-type fructan prebiotics (SMD: –0.34; 95% CI: –0.66–0.01; *p* = 0.04), while inulin-type fructans worsened flatulence (SMD: 0.85; 95% CI: 0.23–1.47; *p* = 0.007). Prebiotics increased absolute abundance of Bifidobacteria (WMD: 1.16 log10 copies of the 16S ribosomal RNA gene; 95% CI: 0.06–2.26; *p* = 0.04).	IBS-D IBS-C IBS-M IBS-U
Yasukawa et al. (2019) [151]	Prebiotics (partially hydrolyzed guar gum)	Double-blind, placebo-controlled, parallel RCT	44	BSS was significantly normalized in the PHGG group compared to placebo. Fecal microbiome analysis using 16S rRNA detected significant changes in the ratios of some bacteria populations between the groups, including a higher level of Bifidobacterium detected in the PHGG group (*p* < 0.05)	IBS-D
Barboi et al. (2022) [154]	Prebiotics (inulin-based)	Randomized cross-over case-control study	47	Abdominal pain severity improved by 68.3% after diet and prebiotics (*p* = 0.004), and abdominal bloating severity parameter improved by 34.8% (*p* = 0.04). The stool number per week and the stool consistency according to the Bristol scale were improved, but without statistical significance between groups (*p* > 0.05).	IBS-C
Cappello et al. (2013) [155]	Symbiotic mixture	Double-blind, placebo-controlled RCT	64	Responders for abdominal bloating were 46.9% in the symbiotic group and 65.6% in the placebo group (*p* = 0.21), and, for flatulence, 50% in the symbiotic group and 62.5% in placebo group (*p* = 0.45). Flatulence score was significantly lower with the symbiotic mixture vs. placebo according to the week-by-week comparisons during treatment (ANCOVA, *p* = 0.038). No significant differences between the groups were found for symptoms of bloating, pain, and urgency.	IBS-D IBS-C IBS-M IBS-U
Bogovic Matijašić et al. (2016) [157]	Synbiotic fermented milk	Double-blind, placebo-controlled multicentric	30	Symbiotic product had a time-limited effect in increasing levels of Lactobacillus La-5-like strains and Bifidobacterium animalis ssp. lactis based on analysis of fecal samples. Both the synbiotic product and placebo were also found to temporarily boost levels of Streptococcus thermophilus in stool.	IBS-C
Azpiroz et al. (2016) [161]	Dietary supplementation with scFOS	Randomized, double-blind, placebo-controlled trial	79	Increase in Bifidobacteria in the scFOS-supplemented group (*p* < 0.05), while total anaerobes and most other bacterial groups were not modified. The change in Bifidobacteria in the scFOS group was not statistically different than placebo. The change of Bifidobacteria was +0.6log with scFOS and −0.04log with placebo in the IBS-C patients.	IBS-C
Compare et al. (2017) [160]	Lactobacillus casei DG and its postbiotic	Retrospective monocentric	20	Postbiotic effectively reduced IL-1α, IL-6, and IL-8 mRNA levels in both colonic (*p* < 0.0001, *p* < 0.0001, and *p* < 0.0001, respectively) and ileal mucosa (*p* < 0.0001, *p* < 0.0006, and *p* < 0.0001, respectively). In contrast, IL-10 mRNA levels significantly increased in both ileal and colonic mucosa (*p* < 0.0001 and *p* < 0.0001, respectively).	IBS-D
**FECAL TRANSPLANTATION**					
Johnsen et al. (2018) [167]	FMT via colonoscope	Double-blind, placebo-controlled, parallel-group RCT	87	Specifically, 36 out of 55 individuals (65%) receiving the active intervention showed a response, compared to 12 out of 28 (43%) in the placebo group (*p* = 0.049). Response was defined as symptom relief of more than 75 points assessed by IBS-SSS at 3 months from FMT.	IBS-D IBS-M
Xu et al. (2019) [172]	FMT	Systematic review and meta-analysis	254 (4 studies)	Single-dose FMT using colonoscopy and nasojejunal tube in comparison with autologous FMT as placebo (NNT = 5, RR = 1.59; 95% CI 1.06–2.39; I = 0%) was more effective, and a reduction in improvement of multiple-dose capsule FMT RCTs was registered.	IBS-D IBS-C IBS-M
Ianiro et al. (2019) [173]	FMT	Systematic review and meta-analysis	267 (5 studies)	The studies found that FMT using donor stool via colonoscopy was more effective than autologous stool based on the results of two pooled RCTs (RR = 0.63; 95% CI 0.43–0.93). One trial also suggested FMT from donor stool administered through a nasojejunal tube may be better than autologous stool (RR = 0.69; 95% CI 0.46–1.02).	IBS-D IBS-C IBS-M
El-Salhy et al. (2020) [166]	FMT via EGD	Double-blind, placebo-controlled RCT	165	It was found that 23.6% of patients who received placebo had response, while 76.9% (*p* < 0.0001) of patients who received 30 g FMT and 89.1% (*p* < 00.0001) who received 60 g FMT had a response. Intestinal bacterial profiles also changed significantly in the groups that received FMT	IBS-D IBS-C IBS-M
Wang et al. (2023) [169]	FMT	Systematic review and meta-analysis	516 (19 studies)	A single-stool FMT led to significant decreases in IBS-SSS scores at 1 month (SMD = −65.75, 95% CI −129.37, −2.13), 3 months (SMD = −102.11, 95% CI −141.98, −62.24), 6 months (SMD = −84.38, 95% CI −158.79, −9.97), 24 months (SMD = −110.41, 95% CI −145.37, −75.46), and 36 months (SMD = −104.71, 95% CI −137.78, −71.64)	IBS-D IBS-C IBS-M IBS-U
**DIET**					
Rao et al. (2015) [192]	Low-FODMAP Fibers	Systematic review	381 249	Fibre was beneficial in 3/3 studies. FODMAP-restricted diet improved overall IBS symptoms in 4/4 studies	IBS-C
So et al. (2022) [187]	Low-FODMAP	Systematic review and meta-analysis	403 (9 trials)	Low-FODMAP diet decreased levels of Bifidobacteria compared to controls based on statistical analysis (SMD = −0.25; *p* = 0.2), but found no clear effects on microbiome diversity or other taxa. No differences were found in total or individual fecal SCFAs or pH between the low-FODMAP diet and control diets.	IBS-C IBS-D IBS-M

RCT = randomized controlled trial; SMD = standardized mean difference; CI = confidence interval; IBS-D = irritable bowel syndrome—diarrhea; IBS-C = irritable bowel syndrome—constipation; IBS-M = irritable bowel syndrome—mixed; ANCOVA = analysis of covariance; PHGG = partially hydrolyzed guar gum; scFOS = short-chain fructo-oligosaccharides; FMT = fecal microbiota transplantation; EGD = esophagogastroduodenoscopy; FODMAP = fermentable oligosaccharides, disaccharides, monosaccharides, and polyols.

## 6. Conclusions and Future Perspectives

IBS is a common multifactorial functional gastrointestinal disorder. Despite its benign nature, it remains challenging to manage and treat adequately.

Emerging evidence over recent decades reveals intestinal microbiota changes in IBS patients. While the causal link between IBS and dysbiosis remains unclear, dysbiosis may promote IBS pathogenesis through metabolic dysfunction, barrier disruption, immune activation, and brain–gut axis disturbances. Elucidating compositional features of the gut microbiota is an initial critical step in delineating the mechanisms of dysbiosis in IBS, providing key insights into disease pathogenesis and enabling targeted therapies.

Therapeutic modulation of the microbiota may improve clinical outcomes in IBS patients. The evidence examined in this review highlights the key role of gut microbiota in both IBS-D and IBS-C features while laying the foundation for novel research directions into specific therapeutic microbiota manipulation strategies for each peculiar subgroup, as supported by the microbiota differences evidenced. In this context, the use of different therapeutic tools, such as prebiotics, probiotics, synbiotics, postbiotics, and also fecal transplants, need to be deepened in order to establish their role in the upcoming years, as new evidence points the way toward increasingly effective treatments, especially for some of these, while, for others, conflicting or biased results have emerged. Particularly concerning probiotics, data from the literature show great heterogeneity and no univocal agreement on their clinical application in the IBS setting [195].

Multi-omics analysis is enabling detailed IBS characterization and the development of precision approaches to personalized treatment. In this context, recent advances in various “omics” technologies have enhanced our ability to characterize the complex interplay between the gut microbiome and human host. Integrated analyses of metataxonomic, metagenomic, metabolomic, genomic, epigenomic, transcriptomic, and proteomic data may help elucidate the biological networks underlying microbiome–intestinal interactions. Multi-omics research has started to uncover novel pathways and microbe–enteric relationships potentially relevant to IBS pathophysiology. The further leveraging of such discovery approaches could facilitate the identification of biomarkers with diagnostic or therapeutic potential. 

Several emerging microbiota-directed therapeutic approaches warrant further exploration for IBS. These include engineered probiotic bacterial strains, bacteriophage therapy, stem cell microfluidic intestine-on-a-chip models, and CRISPR-Cas9 gene editing of the gut microbiota [196,197]. By targeting specific dysbiotic microbes or their effects, such innovative strategies may translate advances in IBS pathophysiology into microbiota-mediated clinical management. Finally, the role of the viroma and mycobioma remains a field almost entirely to be explored [198].

## Figures and Tables

**Table 1:** Principle pathogenetic mechanisms in IBS in which the microbiota is involved.

Mechanism	Description
Impaired gut barrier	Disruption of the intestinal epithelial barrier integrity allows increased gut permeability, permitting translocation of microbes and microbial components across the epithelium. This exposure of the mucosal immune system to luminal microbes and antigens provokes aberrant inflammatory responses that mediate symptoms. Structural and functional defects of tight junction proteins contributing to this barrier impairment have been described in IBS [8,9,10,42].
Aberrant immune response	Intestinal mucosa in IBS patients exhibits immune activation, detectable through increased production of pro-inflammatory cytokines. This mucosal inflammation may occur due to direct immune stimulation from translocated microbes or indirect activation by microbial antigens. Immune responses appear skewed towards pro-inflammatory Th1 and Th17 pathways in IBS. Visceral hypersensitivity, i.e., heightened pain perception from the bowel, is also associated with mucosal immune activation, contributing to abdominal pain symptoms [48,49,50,51,75].
Molecular mimicry	Molecular mimicry between microbial antigens and host proteins can prompt cross-reactive immune responses due to sequence or structural homology between a bacterial epitope and self-antigen. This bacteria–host mimicry induces autoimmune reactions targeting host cells and tissues, thereby perpetuating inflammation and tissue damage. Antibodies formed against bacterial cytolethal distending toxin B, for example, can cross-react with the host protein vinculin and disrupt intestinal nerve function [58,61,62].
Brain–gut axis	The gut microbiota interacts bidirectionally with the central nervous system and enteric nervous system through neuronal, endocrine, and immune signaling pathways in the gut-microbiota–brain axis. Microbial metabolites such as short-chain fatty acids can modulate neurotransmission or induce epigenetic changes that alter nerve signaling. Stress can also change microbiota composition and function through effects on gastrointestinal motility, secretions, and epithelial permeability mediated by the hypothalamic–pituitary–adrenal axis [65,66,67,68,69,70].
Epigenetic changes	Microbial metabolites, especially short-chain fatty acids like butyrate, can induce epigenetic changes by inhibiting histone deacetylases. This inhibition causes histone hyperacetylation, thereby regulating chromatin structure and gene transcription. By modifying epigenetic processes controlling host gene expression, microbial metabolites may contribute to intestinal and neural changes underlying IBS [73,74].

## Data Availability

Not applicable.
